# ID 129 - Recomendações de Tratamento Farmacológico do SUS em Relação ao Cenário Internacional: onde estamos?

**DOI:** 10.5327/2237-9622.2025.v34s1.129

**Published:** 2025-11-25

**Authors:** Andréa da Silva Dourado, Clara Regina Paschoali Martino, Daniela Oliveira de Melo

**Keywords:** protocolos clínicos, guia de prática clínica, condições clínicas, avaliação de tecnologias em saúde

## Abstract

**Introdução:**

Protocolos Clínicos e Diretrizes Terapêuticas (PCDT) são documentos normativos que estabelecem critérios para o cuidado de condições em saúde no âmbito do Sistema Único de Saúde (SUS). Em contrapartida, Diretrizes Clínicas (DC) são elaboradas em caráter norteador para orientação de prática clínica em diferentes contextos de saúde. Em ambos os casos, esses documentos e a publicação deles visam à otimização do cuidado dos pacientes. Nesse sentido, este estudo teve como objetivo comparar recomendações sobre tratamento farmacológico previstas em PCDT e DC internacionais.

**Método:**

A busca por PCDT foi realizada em sítios eletrônicos oficiais do governo federal em 3 de novembro de 2022, sendo incluídos aqueles documentos que possuíam lista formal de recomendação. A busca por DC referentes às condições clínicas abordadas nos PCDT incluídos foi realizada nos repositórios: National Institute for Health and Care Excellence, Scottish Intercollegiate Guidelines Network, Veterans Affairs and the Department of Defense e Ministerio de Salud del Chile. As recomendações sobre tratamento farmacológico de PCDT e DC foram extraídas e comparadas. Realizou-se análise descritiva dos resultados, com ênfase na conflitos e/ou diferenças entre as recomendações.

**Resultados:**

Os 101 PCDT incluídos forneciam 538 recomendações sobre tratamento farmacológico. Foram identificadas 83 DC com 501 recomendações para o manejo farmacológico de 36 (35,6%) das condições abordadas em PCDT. Não foram identificadas recomendações conflitantes entre os documentos. Porém, para 30 (83,3%) doenças haviam diferenças no tratamento priorizado: (1) para 21 condições (58,33%) haviam medicamentos recomendados apenas em PCDT; (2) para 28 (93,3%) condições haviam recomendações em DC não reproduzidas nos PCDT. As condições clínicas com maior número de recomendações ausentes em PCDT foram epilepsia (n=23) e asma (n=11) e aquelas com maior número de recomendações ausentes em DC foram dislipidemia (n=6) e lúpus eritematoso sistêmico (n=5).

**Conclusão:**

Apesar da ausência de conflitos entre recomendações sobre tratamento farmacológico previstas em PCDT e DC, foram observadas diferenças nestes documentos em relação à priorização de medicamentos para o tratamento da maioria das condições clínicas avaliadas. A quantidade de condições para as quais não constavam em PCDT os medicamentos recomendados em cenário internacional pode ser explicada pelo processo de atualização de PCDT, ou por questões que envolvem a disponibilização do medicamento no SUS - registro sanitário e etapas para avaliação de incorporação. Deve-se considerar que, devido a mudanças históricas na concepção, regulamentação e elaboração de PCDT, a lista formal de recomendações de fármacos pode não refletir a totalidade de tecnologias disponíveis no SUS, contribuindo para o aumento do número de condições com diferenças nas linhas de cuidado. Compreendendo PCDT como políticas públicas, espera-se que sejam específicos ao contexto. Nesse sentido, a ausência de recomendações semelhantes de PCDT em DC pode indicar necessidades nacionais diante das características de organização e/ou medicamentos disponibilizados no SUS.

